# Screening parents of children with a chronic condition for mental health problems: a systematic review

**DOI:** 10.1136/archdischild-2024-328300

**Published:** 2025-02-20

**Authors:** Nadia Coscini, Grace McMahon, Madison Schulz, Casey Hosking, Melissa Mulraney, Anneke Grobler, Harriet Hiscock, Rebecca Giallo

**Affiliations:** 1 Department of Paediatrics, The University of Melbourne, Parkville, Victoria, Australia; 2 Health Services Group, Murdoch Children’s Research Institute, Parkville, Victoria, Australia; 3 Intergenerational Health Group, Murdoch Children's Research Institute, Parkville, Victoria, Australia; 4 Neurodisability and Rehabilitation Group, Murdoch Children's Research Institute, Parkville, Victoria, Australia; 5 Murdoch Childrens Research Institute, Parkville, Victoria, Australia; 6 Department of Paediatrics, Deakin University School of Psychology—Geelong Waterfront Campus, Geelong, Victoria, Australia

**Keywords:** Mental health, Child Health, Health Care Economics and Organizations, Health services research, Paediatrics

## Abstract

**Objective:**

Parents of children with a chronic condition (CC) have a high prevalence of mental health (MH) difficulties. It is not known whether establishing screening programmes in paediatric clinics to identify parental MH difficulties increases detection or referrals to support services. We aimed to identify approaches to routine screening programmes for parents of children with a CC attending hospital outpatient clinics (aim 1); associated prevalence of MH symptoms (aim 2); and whether screening impacted referrals to, and uptake of, MH services (aim 3).

**Design:**

Medline, Embase, PsycINFO, CINAHL and PubMed databases were searched between January 2000 and December 2023. Studies were selected if they conducted routine screening of MH of parents of children with CCs (aged <18 years). Study characteristics, population demographics and information on screening tools, MH symptoms and referral pathways were extracted.

**Results:**

Eight articles met the inclusion criteria from 8673 screened. The prevalence of elevated parental MH symptoms ranged between 9.6% and 62.9% for anxiety and 7.7% and 57.0% for depression. Two studies using the Distress Thermometer for Parents found 3.3%–57.0% had elevated levels of ‘clinical distress’. There was limited detail on referral pathways, referrals made and uptake.

**Conclusions:**

Elevated MH symptoms are common in parents of children with CCs, but there is wide variability in outcomes. More research is required to understand this and how best to identify and screen for and support parents with referrals to and uptake of services for their MH.

**PROSPERO registration number:**

CRD42023438720.

**WHAT IS ALREADY KNOWN ON THIS TOPIC**

- Parents of children with chronic conditions (CCs) have high rates of mental health (MH) difficulties, such as anxiety and depression.
- There is one set of international guidelines for screening the MH of parents of children with cystic fibrosis (CF) annually with recommended referral pathways and one systematic review specifically for anxiety in children and parents of children with CF.

**WHAT THIS STUDY ADDS**

- This systematic review considers systematic parental MH screening across a wider range of CCs and confirms high prevalence of MH conditions but highlights limited details on the impact of screening on rates of referrals and their uptake by parents.

**HOW THIS STUDY MIGHT AFFECT RESEARCH, PRACTICE OR POLICY**

- More research is needed on consensus around the administration of universal MH screening, including codesign with parents and healthcare providers.
- Longitudinal data are required to demonstrate whether routine parental MH screening leads to uptake of referral services, and, in turn, improved MH for those parents experiencing MH difficulties.

## Introduction

 Chronic conditions (CCs) are defined as long-term illnesses that have ongoing sequelae and include conditions such as asthma, kidney disease and diabetes.^1^ More than 40% of children in Australia have one CC and one in five children has more than two CCs.^1^ With rates of CCs steadily growing^2^ and improved medical care and technology, the number of children surviving longer with CCs that were previously life-limiting is also increasing.^3^ Having a child with a CC is stressful for families due to the demands of caregiving, medical management, financial difficulties, appointments and the emotional needs of family members.^4^

A recent cross-sectional study by Thomas *et al* surveyed 194 parents of children with diabetes, asthma, congenital heart disease (CHD) and cancer using the Depression, Anxiety and Stress Scale and reported anxiety in 38% of parents, depression in 26% and stress in 40%.^5^ This is in contrast to the prevalence of depression and anxiety in adults in Australia’s National Study of Mental Health and Wellbeing (2020–2022), where anxiety in adults (16–85 years of age) was reported at 17.2% and 7.5% for a mood disorder.^6^ A recent international meta-analysis of 26 studies investigating the parent mental health (MH) of children with CCs found that 35% of parents scored above the cut-off thresholds for depression symptoms and 57% for anxiety symptoms on standardised screening measures.^7^ Addressing poor parental MH is essential not only for parent functioning but also for the mental and physical health of children who are at increased risk of psychological difficulties^8^ and lower quality of life.^7^ In this review, we refer to screening as routinely asking parents about MH symptoms through using measures such as those identifying anxiety, depression and stress.

Identifying and supporting parents of children with CCs experiencing poor MH may have the potential to significantly improve child and family well-being. The International Committee on Mental Health in Cystic Fibrosis (ICMHCF) has published evidence-based guidelines recommending annual MH screening in children with cystic fibrosis (CF) and their caregivers.^9^ However, other than these guidelines, we could find no other recommendations for systematic screening of MH in parents of children with CCs. Therefore, the aims of this study were to identify: (1) approaches to routine screening programmes for parents of children with CCs attending hospital outpatient clinics; (2) associated prevalence of MH conditions found in screening programmes and (3) whether screening programmes impact referrals to and uptake of MH services.

## Methods

### Design

We conducted a systematic review per the Preferred Reporting Items for Systematic Reviews and Meta-analyses statement.^10^ The protocol was registered with the PROSPERO International Register of Systematic Reviews on 3 July 2023 (CRD42023438720).

### Search strategy

We searched five databases: Medline, Embase, PsycINFO, CINAHL and PubMed. The search included articles published between 1 January 2000 and 31 December 2023. No language restrictions were applied. Key search terms included variations of “chronic disease”, “caregiver”, “parent”, “mental health”, “screen”, or “measurement” (see online supplemental file 1 for details).

### Literature search

Included studies had (1) parents of children with a mean age <18 years who had a CC, (2) parents who were part of a systematic screening process with a validated MH or stress measure in an outpatient clinical setting and (3) validated measures of MH or stress that reported prevalence. We referred to systematic or routine screening as screening of all caregivers (as opposed to a selected group) was either in existence in the clinic or being established with a long-term aim, where the study may also have been part of an evaluation. Studies were excluded if they: (1) included screening parents of young adults with a mean age >18 years, (2) included CCs that were life-limiting, with neurodisability or neurological decline, were psychiatric, surgical or part of an acute medical crisis period, (3) did not use a validated measure of MH or stress, (4) were not part of an established programme of consecutive screening of parents during routine clinic appointments or (5) were not conducted in countries included in the top 50 United Nations Human Development Index (2020) report.^11^ Restricting the review to the top 50 countries leads to homogeneity around comparable healthcare systems.^12^ The only country where we identified studies not in the top 50 was Türkiye, and it was excluded.

### Data extraction

Each article was screened by two independent authors. This screening was performed by NC and one of GM, MS, CH and RG. Titles and abstracts of search results were screened using Covidence^13^ software, with disagreements resolved by discussion with the primary author (NC). Two authors (NC and RG) independently reviewed the full text of the included articles to determine their final inclusion, and any conflicts were resolved by discussion. Data were extracted independently from the included studies on a standardised study-designed table (NC and RG). We collected data on the study participants, screening details and the referral process.

### Quality assessment

Two authors (NC and RG) independently assessed included studies for quality using the standardised Joanna Briggs Institute critical appraisal checklist for cross-sectional studies reporting prevalence data.^14^ Results were recorded as ‘yes’, ‘no’ or ‘unclear’, and any disagreements were resolved by discussion between these two authors.

## Results

### Selected studies

**Figure 1:**
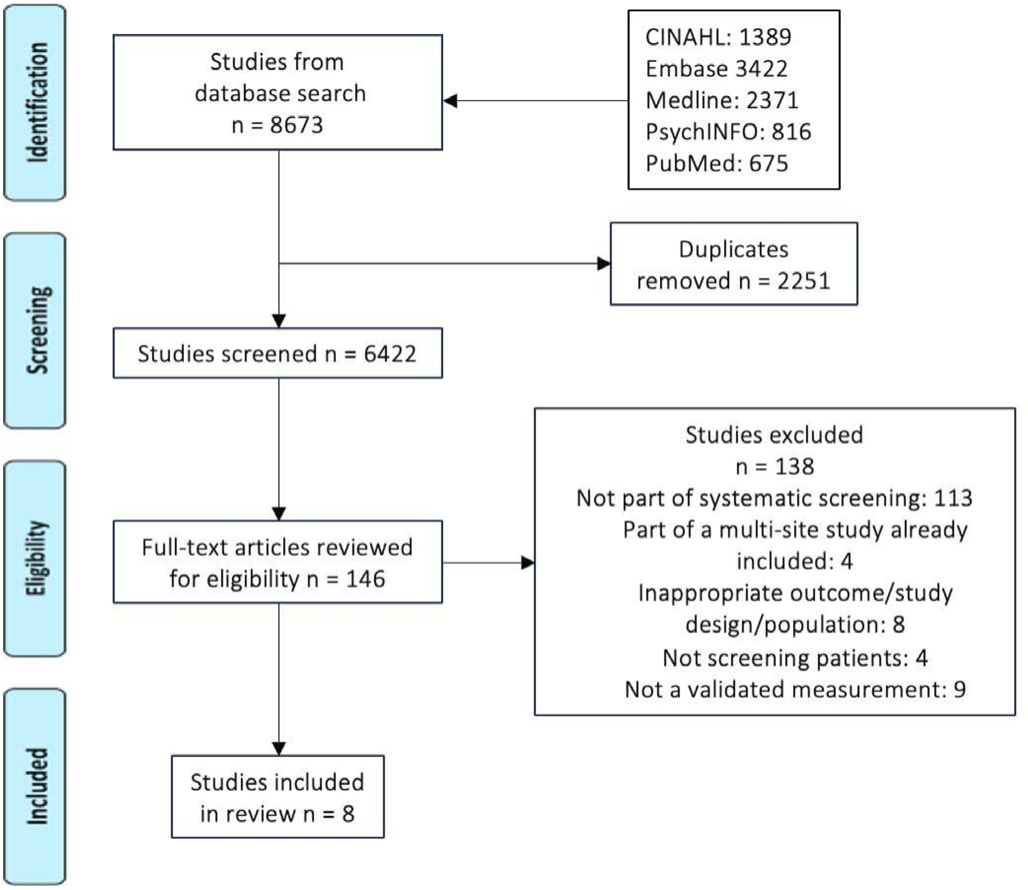
PRISMA flow diagram. PRISMA, Preferred Reporting Items for Systematic Reviews and Meta-Analyses.

Figure 1 demonstrates the PRISMA flow diagram with the initial search yielding 8673 articles with 146 articles retrieved for full-text review. Of these, eight articles met the inclusion criteria. Four additional studies were reviewed,^15^,^18^ which were part of The International Depression/Anxiety Epidemiological Study (TIDES)^19^ of 4102 caregivers of a child with CF across nine countries. As they were already incorporated into a larger multisite study,^19^ they were excluded from the final eight articles. A meta-analysis was not undertaken due to the small number of studies and heterogeneity of interventions and outcome measurements. We have, therefore, reviewed outcomes in a narrative summary and present data in tables1  2 to allow for cross-comparison.

**Table 1:** Description of included studies

First author, year, country	Study design	Medical condition	Total (n) and % female		Mean parent age (years)	Parent university education (%)	Screening measure(s)	Anxiety, overall prevalence %, (%, 95% CI by gender)		Depression, overall prevalence %, (%, 95% CI by gender)		Other: overall prevalence %, (%, 95% CI by gender)	
								Female	Male	Female	Male	Female	Male
Diederen^24^ 2018 Netherlands	CS, prospective	IBD	n=87 92%		45.3	42.5	DT-P					Clinical distress: 47.0	
												Not reported	
Graziano^26^ 2020 Italy	CS, prospective	CF	n=186 63%		Not reported	44	GAD-7 PHQ-9	62.9 (56, 70)		57.0 (50, 64)		Suicidality	
								65.8 (59, 73)	58.0 (51, 65)	61.5 (54, 68)	49.3 (42, 56)	7.0 (4, 12)	0.0 (0, 2)
Graziano^20^ 2023 Italy	CS, retrospective	PCD, CF	n=129 56%		PCD: 45.9	Not reported	GAD-7 PHQ-9	PCD:		PCD:		Suicidality—PCD	
								54.2 (46, 63)	47.4 (39, 56)	54.2 (46, 63)	42.1 (34, 51)	20.0 (14, 28)	10.0 (6, 16)
					CF: 45.7			CF:		CF		Suicidality—CF:	
								56.2 (48, 64)	39.5 (31, 48)	45.8 (37, 54)	44.7 (36, 53)	5.0 (2, 10)	3.0 (1, 7)
Lee^23^ 2022 USA	CS, retrospective	CHD	n=281 44%		Not reported	Not reported	DASS-21					Stress	
			1 year	n=173				13.5 (9, 19)	5.6 (3, 10)	5.9 (3, 10)	1.9 (1, 5)	11.8 (7, 17)	5.6 (3, 10)
			2 years	n=98				11.7 (7, 20)	0.0 (0, 4)	7.5 (4, 14)	0.0 (0, 4)	17.0 (11, 26)	2.9 (1, 8)
			3 years	n=134				4.4 (2, 9)	2.2 (1, 6)	3.3 (1, 8)	4.7 (2, 10)	5.5 (3, 11)	0.0 (0, 3)
Quittner^19^ 2014 nine countries*	CS, multi-site‡, prospective	CF	n=4102 76%		Not reported	Not reported	HADS CES-D	44.8 (43, 46)		31.6 (30, 33)			
								47.8 (46, 49)	35.2 (34, 37)	33.8 (32, 35)	24.6 (23, 26)		
Vassilopoulos^21^ 2021 USA	CS, retrospective	CF	n=104 76%		Not reported	Not reported	GAD-7 PHQ-9	9.6 (5, 17)		7.7 (3, 15)			
								Not reported		Not reported			
Verkleij^25^ 2018 Netherlands	CS, prospective	CF	n=79 58%		41	Not reported	GAD-7 PHQ-9	Combined screening clinically elevated score: 13.9 (7, 24) Female 7.6 (2, 16) Male 3.8 (1, 11)				Suicidality	
								Screening and psychological assessment: 40.5 (30, 52) Female 27 (17, 38) Male 13.9 (7, 24)				3.0 (0, 10)	1.0 (1, 6)
Warnink-Kavelaars^22^ 2021 Netherlands	CS, unmatched case–control†	MS	n=67 64%		41.2	55	DT-P					Clinical distress: 31.3	
												33.3 (23, 45)	28.0 (19, 40)

* Nine countries: Belgium, Germany, Italy, Spain, Sweden, The Netherlands, Türkiye, UK and USA. However, due to small sample size (n=52), the authors excluded Türkiye in final data analysis.^19^

† 67 parents were compared with 1134 parents in a Dutch control group who were part of broader DT-P validation study.^28^

‡ All studies were single-centre unless otherwise indicated.

CES-D, Center for epidemiological studies depression; CF, cystic fibrosis; CHD, congenital heart disease; CS, cross-sectional; DASS-21, Depression, Anxiety and Stress Scale 21-item; DT-P, Distress Thermometer for Parents; GAD-7, Generalised Anxiety Disorder assessment; HADS, Hospital Anxiety and Depression Scale; IBD, inflammatory bowel disease; MS, Marfan syndrome; PCD, primary ciliary dyskinesia; PHQ-9, Patient Health Questionnaire (depression scale).

**Table 2:** Screening measure process and subsequent support

Study	Administering the screening measure						Support or services provided		
	Preclinic	At annual clinic visit	Online	By clinician	Scorer	Response rate	Psychology/allied health review	Referred to services	Details
Diederen *et al*^24^	✔	✔	✔	Not reported	Not reported	Not reported	Not reported	Not reported	Not reported
Graziano *et al*^26^	Not reported	✔	Not reported	Not reported	Psychologist	Not reported	For suicidal parents	✔	Psychoeducation, psychotherapy, pharmacology
Graziano *et al*^20^	Not reported	✔	Not reported	Not reported	Psychologist	100%	Not reported	✔	Psychoeducation, psychotherapy, pharmacology
Lee and Loomba^23^	Not reported	✔	Not reported	Not reported	Not reported	Not reported	Not reported	Not reported	Not reported
Quittner *et al*^19^	Not reported	✔	Not reported	Not reported	Varied	Not reported	Not reported	✔	‘As per CF guidelines’
Vassilopoulos*et al*^21^	Not reported	Not reported	Not reported	Not reported	Not reported	Not reported	On the team but no details	✔	Community MH
Verkleij *et al*^25^	Not reported	✔	Not reported	Trained staff/nurse	Not reported	Not reported	Yes*	✔	Internal social worker, psychologist±family coach
Warnink-Kavelaars *et al*^22^	✔	✔	✔	Not reported	Not reported	57%	Not reported	Not reported	Not reported

* Psychologist also completed an interview after screening, but intervention was provided mostly via a social worker for caregivers.

CF, cystic fibrosis; MH, mental health.

### Study characteristics

Table 1 summarises the characteristics of the included studies. A total of 5035 parents were represented. Seven of the eight studies were cross-sectional (two with retrospective components,^20 21^ one an unmatched case control^22^ and one study was based on retrospective chart review over multiple timepoints).^23^ Three studies were based in the Netherlands,^22 24 25^ two in Italy,^20 26^ two in the USA,^21 23^ and one involved nine countries (Belgium, Germany, Italy, Spain, Sweden, The Netherlands, Türkiye, the UK and the USA).^19^ Five studies assessed parents of children with CF^19 21 25 26^ and one with CF or Primary Ciliary Dyskinesia (PCD).^20^ The final three studies screened parents of children with inflammatory bowel disease,^24^ Marfan syndrome^22^ and CHD,^23^ respectively. Parent education levels were only reported in three studies, showing that 44%–74% of parents had completed university.^22 24 26^

### Aim 1: approaches to routine screening for elevated MH symptoms among parents

All studies used brief screening measures (see table 1), and participants in one study also completed a 45-min interview with a psychologist.^25^ Table 2 summarises the screening processes. Only two studies mention that the screening measure was completed before the clinic visit; both used a national online portal programme.^24 25^ In all but one study,^21^ screening occurred during annual clinic visits, the other measured MH serially every 6 months. In one study, a trained staff member administered and scored the screening measure,^25^ while in two studies, a psychologist/other health professional reviewed the scores.^19 26^ Only two studies reported their screening rate of parents (57%^20^ and 100%,^24^ respectively). Two studies used the Distress Thermometer for Parents (DT-P),^22 24^ which was adapted from the Distress Thermometer for adults and was validated in the Netherlands^27^ where both these studies occurred. The screening tool combines six areas of concern: (emotional, physical, social, practical, cognitive and parenting) and provides an overall ‘distress’ score between 0 and 10.^24^ Based on validated normative data from a large-scale Dutch study,^28^ scores above a cut-off of 4 are categorised as ‘clinical distress’, which we identified as an MH symptom.

### Aim 2: prevalence of elevated MH symptoms arising from screening

Prevalence of elevated anxiety symptoms ranged between 7.6% and 62.9%, and prevalence of elevated depressive symptoms ranged between 7.7% and 57.0%. Three studies reported on suicidality with prevalence ranging between 1% and 20%, the highest prevalence seen in parents of children with PCD.^20 25 26^ Verkleij combined the MH scores for the Generalised Anxiety Disorder assessment (GAD-7) and Patient Health Questionnaire-9 (PHQ-9), reporting that 13.9% of parents had elevated combined scores.^25^ All the participants in this study also completed a diagnostic psychological assessment (with a face-to-face interview with a psychologist or social worker) irrespective of their screening results, and a further 40.5% (n=32) had concerns identified regarding clinically elevated levels of anxiety and depression. Vassilopoulos *et al*^21^ report a prevalence of 8.3% for anxiety symptoms and 6.6% for depressive symptoms. In the studies that reported on prevalence by gender, all found higher rates of elevated symptoms in mothers than fathers (see table 1). Stress was also reported across multiple ages in one study for parents of toddlers with CHD, with levels peaking at 2 years of age.^23^

### Aim 3: impact of screening on rate of referrals to (and uptake of) services by parents at risk of an MH disorder

Table 2 summarises the reported referral pathways for each study and referral details. Verkleij *et al* was the only study that reported that all individuals with clinically elevated scores were referred for further support services (63.6% accepted this referral) and the length of subsequent intervention (14 of 38 referrals to a social worker continued treatment for more than 12 months).^25^

### Quality assessment

There was a moderate risk of bias in six studies, with a further two studies having a high risk of bias (table 3).^20 21^ All studies demonstrated an appropriate sample frame and the use of validated assessments to identify elevated symptoms of anxiety, depression, stress and clinical distress. However, there was a general lack of detail about how screening occurred^21 22^ and the proportion of parents who completed screening measure(s).^20^,^2224^

**Table 3:** Quality assessment: JBI critical appraisal checklist for cross-sectional studies

Study	Appropriate sample frame?	Appropriate participant sampling?	Adequate sample size?	Adequate subjects and setting detail?	Sufficient coverage in data analysis?	Valid assessments to identify the condition?	Standard way to measure a condition?	Appropriate statistical analysis?	Adequate response rate?
Diederen *et al*^24^	Yes	Yes	Unclear	Yes	Yes	Yes	Yes	Yes	Unclear
Graziano *et al*^26^	Yes	Yes	Yes	Yes	Yes	Yes	Yes	Yes	Yes
Graziano *et al*^20^	Unclear	Unclear	No	Unclear	Unclear	Yes	Yes	Yes	Unclear
Lee and Loomba^23^	Yes	Yes	Yes	Yes	No	Yes	Yes	Yes	Yes
Quittner *et al*^19^	Yes	Yes	Yes	Yes	Yes	Yes	Yes	Yes	Yes
Vassilopoulos *et al*^21^	Yes	Unclear	Unclear	No	Unclear	Yes	Unclear	Yes	Unclear
Verkleij *et al*^25^	Yes	Yes	Yes	Yes	Yes	Yes	Yes	No	Yes
Warnink-Kavelaars *et al*^22^	Yes	Yes	No	Yes	Yes	Yes	Unclear	Yes	Unclear

JBI, Joanna Briggs Institute.

## Discussion

This is the first review examining outpatient screening rates for MH difficulties, along with subsequent referral and service uptake patterns, in a cohort of parents of children with CCs. There was considerable variation in screening outcomes across the studies and across different types of CCs, which is consistent with the existing research.^7 29^ This variability suggests that findings may have limited generalisability across different disease contexts. While the review provided valuable insights, several limitations were evident. The analysis was constrained by the small number of included studies, many of which shared one large sample, preventing a comprehensive meta-analysis. Five of the eight studies focused specifically on CF, with four using identical screening instruments, which allowed for some comparative analysis. Additionally, the predominance of cross-sectional studies and the lack of data on screening completion rates and service uptake made it difficult to determine whether screening programmes improve the detection and treatment of parental MH difficulties compared with standard care.

### Approaches to routine screening for parental MH

There was no consistent approach to screening, with limited detail on how screening assessments were delivered, completed and scored. It also raises questions about response accuracy and potential impact on the heterogeneity of outcomes we found. The general paucity of data around the administration process of universal screening for MH is consistent with the findings of a 2013 systematic review on screening for paediatric MH in hospital clinics, which looked at 38 studies.^30^ The review found that there was limited information about how families were approached about screening, the process and its administration.^30^ Even though this focused on paediatric patients rather than parents, there was a similarity in outpatient environments to what we examined. For our systematic review, the exceptions were the studies that using the DT-P,^22 24^ which was incorporated into the child’s KLIK (Kwaliteit van Leven In Kaart) programme for completion. KLIK refers to the ‘Patient-Reported Outcome Measures’ online programme established in the Netherlands in 2011 for children with CCs and their caregivers to complete online quality-of-life questionnaires before clinic appointments.^31^

Salley *et al* argue that when establishing MH care for parents in hospitals those implementing the screening programme must be appropriately trained in both administering the measurement and providing follow-up.^32^ While there was limited information about the specifics of the screening process for the articles on CF, three articles have been published discussing the implementation of the ICMHCF recommendations for CF clinics across Europe, the USA and England.^33^,^35^ They found multiple barriers in establishing routine screening with specific implementation challenges centred around where to accurately record parent data (ie, not as part of the child’s record) and how to provide appropriate follow-up.^34^ More research may be needed to understand best practice for administering screening questionnaires involving caregivers and healthcare providers in the co-design of the administration process.

### Prevalence and wide variation of parental MH symptoms from screening

This review is notable for the heterogeneity in screening outcomes evident in the broad range in prevalence of elevated MH symptoms. This may be influenced by the child’s underlying CC, the screening instrument and when it is applied. Four of the five studies on CF demonstrated similarly high prevalence levels of anxiety and depression symptoms. The Vassilopoulos study was an outlier with low prevalence, which the authors attributed to having access to both psychology in the outpatient clinic and inpatient psychiatry for review and initial intervention.^21^ The study also found that parents with elevated MH symptoms had children with higher numbers of hospital admissions and longer lengths of stay, suggesting higher levels of parental anxiety impact on more health-seeking behaviours, while those with depression may have less capacity for adequate treatment, leading to increased hospital treatment in both cases.^21^ This bidirectional relationship between a child with a CC and parental MH is well documented in the literature.^36^ The interplay between parental MH, and the child’s emotional and physical health outcomes needs to be considered when interpreting the outcomes in screening.

The wide variability of screening outcomes may also be associated with underreporting and a lack of sensitivity of screening instruments. The TIDES study, which used the HADS and CES-D, demonstrated that the HADS underestimated depression rates and had lower sensitivity,^19^ with the subsequent ICMHCF guidelines recommending the PHQ-9 and GAD-7.^9^ Screening questionnaires may also underestimate levels of elevated MH symptoms due to their self-report nature. This raises questions about true prevalence, as parents may underestimate the impact of their child’s illness on their MH. This was most acutely demonstrated in the Verkleij *et al* study where a further 40.5% of parents were assessed as having moderate-to-severe anxiety and/or depression symptoms in a 1–1 semistructured interview with a psychologist after being screened.^25^ The authors proposed parents may have underreported on the screening tests because they wanted to minimise their symptoms with ‘positive thinking’, or perceived that their symptoms were a normal part of life.^25^ Other studies have also shown this pattern where levels of self-reported stress did not correlate with validated assessments or may consider symptoms as part of typical life experience.^37 38^ The variation in study population, individual caregiver perception of MH symptoms and different international contexts may have had an impact on the variation in outcomes noted from screening. Further research with consistent validated measures in large samples in different environments is required to allow for more consistent cross-comparison along with caregiver and clinician input to highlight the most appropriate instrument that may more likely identify areas of difficulty.

The changing rates of stress recorded at different timepoints in Lee’s study of parents of children with CHD not only highlight a role for serial screening but suggest that different timepoints in different disease conditions may contribute to heterogeneity in reported prevalence outcomes. Lee’s study examined depression, anxiety and stress levels at 6 monthly timepoints and while anxiety and stress were high at 1 year of age (usually a few months post-CHD surgery), stress peaked when children were aged 2 years before decreasing at 3 years of age.^23^ A systematic review of parents of children with type 1 diabetes showed high anxiety levels at diagnosis and higher anxiety for those with younger children.^39^ MH symptoms can therefore fluctuate and coupled with the long-term nature of CCs, illness changes or developmental transitions may have an impact on parental MH.^40^ If screening is to be administered, future research should aim to determine whether screening should be offered serially or associated with specific timepoints in the child’s life. Additional consideration should also be given to varying disease severity and illness trajectories across each condition when healthcare services are developing screening approaches that need to adapt to a specific patient population.

Outcomes on suicidal ideation further reflect the wide variability in screening outcomes, possibly associated with the underlying condition. Graziano hypothesised that the higher rate of suicidal ideation in parents of children with PCD^20^ reflected the difficulty of the diagnosis, treatment and support system profile of PCD compared with CF.^20^ Further research is needed to understand this pattern and whether diverse conditions, support structures and other factors contribute to these outcomes. Ultimately, they also highlight the variable and serious nature of the impact on caregiver MH and the need to consider appropriate risk assessment when tailoring screening.

### Impact of screening on the rate of referrals to and uptake of services for parental MH

The lack of data around the number of parents who accessed services (and whether they were accessed or had an impact) makes it difficult to conclude if screening is a useful process. Information provided on referral pathways was largely generic, with only one study providing numbers of parents who accepted referrals to support services.^25^ Overall, more data are needed on whether universal screening for the detection of elevated MH in parents correlates with uptake of referrals and subsequent improvement in MH outcomes. Screening alone may not be sufficient to identify parents and link them into services that provide meaningful improvement.

### Strengths and limitations

The strength of our systematic review includes a broad search strategy with multiple databases, including articles in languages other than English, hand-searched reference lists and use of multiple screeners. The study also had a blinded data extraction process with dual coding. A key strength of our systematic review was the focus on systematic screening of parent MH in real-world clinical settings, as multiple excluded studies used screening only in a cross-sectional research context. Follow-up research on these cohorts may provide important information about the impact of referral pathways on long-term MH outcomes. Knowing if screening has a significant impact would also allow for future research evaluating the cost-effectiveness of early identification and support. Our search strategy also chose to focus on CCs without other factors such as acute crisis. However, this means findings may not generalise to other conditions and further research is required in these populations.

## Conclusion and implications

This review found significant variability in the prevalence of MH symptoms from screening parents of children with CCs within and across multiple conditions. It also demonstrated limited evidence about approaches to systematic screening of MH in hospital outpatient settings. While routine screening may be a useful way to open conversations on symptoms, more research is required to understand the variability in outcomes, and whether diverse clinical populations or screening instruments contribute to this. There was also a lack of data about the number of parents referred to services, whether parents took up these services and if there was any improvement in MH symptoms. Longitudinal data are, therefore, required to demonstrate whether routine parental MH screening leads to uptake of referral services and, in turn, improved MH for those parents experiencing MH difficulties.

## Supplementary material

10.1136/archdischild-2024-328300 online supplemental file 1

## Data Availability

Data are available upon reasonable request. All data relevant to the study are included in the article or uploaded as supplementary information.
