# Supplementary material for: Screening parents of children with a chronic condition for mental health problems: a systematic review
Source: Arch Dis Child. 2025 Feb 20;110(9):e328300. doi: 10.1136/archdischild-2024-328300 (PMC12418540; doi:10.1136/archdischild-2024-328300)
Supplement: online supplemental file 1 [file archdischild-110-9-s001.pdf]

Supplemental File 1. Search strategies by each data library

Embase

1. exp chronic disease/
2. asthma/
3. atopic dermatitis/
4. eczema/
5. congenital heart malformation/
6. insulin dependent diabetes mellitus/
7. exp inflammatory bowel disease/
8. autoimmune disease/ or immune deficiency/
9. exp juvenile rheumatoid arthritis/
10. hemophilia A/
11. hemophilia B/
12. exp endocrine bone disease/
13. ambulatory care/
14. outpatient department/
15. bronchiectasis/
16. cystic fibrosis/
17. chronic kidney failure/ or kidney tubule disorder/ or immediate type hypersensitivity/
18. (asthma or chronic-illness* or dermatitis or eczema or congenital-heart-disease* or congenital-heart-defect* or diabetes-mellitus-Type-1 or diabetes-mellitis-1 or inflammatory-bowel-disease* or crohn* or ulcerative-colitis or juvenile-arthritis or cystic-fibrosis or bronchiectasis or haemophilia or hemophilia or kidney-failure* or immediate-hypersensitivit* or immediate-hyper-sensitivit*).tw,kf,dq.
19. 1 or 2 or 3 or 4 or 5 or 6 or 7 or 8 or 9 or 10 or 11 or 12 or 13 or 14 or 15 or 16 or 17 or 18
20. exp parent/
21. caregiver/
22. legal guardian/
23. (parent* or mother* or maternal or father* or paternal or caregiver* or care-giver* or guardian*).tw,kf,dq.
24. 20 or 21 or 22 or 23
25. mental health/ or psychological well-being/
26. anxiety/ or exp anxiety disorder/
27. exp depression/
28. caregiver burden/ or caregiver burnout/
29. mental stress/ or burnout/
30. (Anxiety or anxious* or depress* or distress or mental-health or stress or caregiver-burden or mental-disorder* or mental-disease*).tw,kf,dq.
31. 25 or 26 or 27 or 28 or 29 or 30
32. (newborn* or new-born* or baby or babies or neonat* or neo-nat* or infan* or toddler* or pre-schooler* or preschooler* or kinder or kinders or kindergarten* or kinder-aged or boy or boys or girl or girls or child or children or childhood or pediatric* or paediatric* or school-age* or schoolage* or schoolchild* or schoolgirl* or schoolboy* or adolescen* or youth or youths or teen or teens or teenage*).tw,kf,dq,hw.
33. (screen* or inventor* or scale* or questio?naire* or assessment* or measurement*).tw,kf,dq.
34. exp questionnaire/
35. mass screening/
36. 33 or 34 or 35
37. 19 and 24 and 31 and 32 and 36

38. limit 37 to dc=20233112-20000101
39.case report/
40. limit 38 to (conference abstract or conference paper or "conference review" or editorial or letter)
41. 38 not (39 or 40)

Medline

1. exp *Chronic Disease/
2. *asthma/ or *asthma, exercise-induced/
3. *dermatitis, atopic/ or *eczema/
4. *Heart Defects, Congenital/
5. *Diabetes Mellitus, Type 1/
6. exp *inflammatory bowel diseases/
7. hemophilia a/ or hemophilia b/
8. *arthritis, juvenile/
9. *bone diseases, endocrine/
10. *ambulatory care/ or *ambulatory care facilities/ or *outpatient clinics, hospital/ or *Patient care management/
11. *bronchiectasis/ or *cystic fibrosis/
12. *kidney failure, chronic/ or *renal tubular transport, inborn errors/ or *hypersensitivity, immediate/
13. (asthma or chronic-illness* or dermatitis or eczema or congenital-heart-disease* or congenital-heart-defect* or diabetes-mellitus-Type-1 or diabetes-mellitis-1 or inflammatory-bowel-disease* or crohn* or ulcerative-colitis or juvenile-arthritis or cystic-fibrosis or bronchiectasis or haemophilia or hemophilia or kidney-failure* or immediate-hypersensitivit* or immediate-hyper-sensitivit*).tw,kf.
14. 1 or 2 or 3 or 4 or 5 or 6 or 7 or 8 or 9 or 10 or 11 or 12 or 13
15. exp *parents/
16. *Caregivers/
17. *Legal Guardians/
18. (parent* or mother* or maternal or father* or paternal or caregiver* or care-giver* or guardian*).tw,kf.
19. 15 or 16 or 17 or 18
20. Mental Health/
21. Anxiety/ or exp Anxiety Disorders/
22. Depression/ or exp Depressive Disorder/
23. caregiver burden/
24. stress, psychological/ or burnout, psychological/
25. (Anxiety or anxious* or depress* or distress or mental-health or stress or caregiver-burden or mental-disorder* or mental-disease*).tw,kf.
26. 20 or 21 or 22 or 23 or 24 or 25
27. (newborn* or new-born* or baby or babies or neonat* or neo-nat* or infan* or toddler* or pre-schooler* or preschooler* or kinder or kinders or kindergarten* or kinder-aged or boy or boys or girl or girls or child or children or childhood or pediatric* or paediatric* or school-age* or schoolage* or schoolchild* or schoolgirl* or schoolboy* or adolescen* or youth or youths or teen or teens or teenage*).tw,kf,hw.
28. (screen* or inventor* or scale* or questio?naire* or assessment* or measurement*).tw,kf.
29. exp "Surveys and Questionnaires"/ or *Mass screening/
30. 28 or 29
31. 14 and 19 and 26 and 27 and 30
32. limit 31 to dt=20233112-20000101
33. limit 32 to (case reports or comment or editorial or guideline or letter or practice guideline)
34. 32 not 33

PsycInfo

1. chronic illness/ or chronic pain/ or chronically ill children/
2. asthma/
3. dermatitis/ or eczema/
4. exp heart disorders/ and congenital disorders/
5. Diabetes Mellitus/
6. ulcerative colitis/
7. hemophilia/
8. arthritis/
9. hypothyroidism/ or hypopituitarism/
10. outpatient treatment/
11. cystic fibrosis/
12. kidney diseases/
13.(asthma or chronic-illness* or dermatitis or eczema or congenital-heart-disease* or congenital-heart-defect* or diabetes-mellitus-Type-1 or diabetes-mellitis-1 or inflammatory-bowel-disease* or crohn* or ulcerative-colitis or juvenile-arthritis or cystic-fibrosis or bronchiectasis or haemophilia or hemophilia or kidney-failure* or immediate-hypersensitivit* or immediate-hyper-sensitivit*).ti,ab,id.
14. 1 or 2 or 3 or 4 or 5 or 6 or 7 or 8 or 9 or 10 or 11 or 12 or 13
15. exp parents/
16. caregivers/
17. (parent* or mother* or maternal or father* or paternal or caregiver* or care-giver* or guardian*).ti,ab,id.
18. 15 or 16 or 17
19. exp mental health/
20. anxiety/ or exp anxiety disorders/
21. "depression (emotion)"/ or exp major depression/
22. caregiver burden/
23. psychological stress/
24. (Anxiety or anxious* or depress* or distress or mental-health or stress or caregiver-burden or mental-disorder* or mental-disease*).ti,ab,id.
25. 19 or 20 or 21 or 22 or 23 or 24
26. (newborn* or new-born* or baby or babies or neonat* or neo-nat* or infan* or toddler* or pre-schooler* or preschooler* or kinder or kinders or kindergarten* or kinder-aged or boy or boys or girl or girls or child or children or childhood or pediatric* or paediatric* or school-age* or schoolage* or schoolchild* or schoolgirl* or schoolboy* or adolescen* or youth or youths or teen or teens or teenage*).ti,ab,id,hw.
27. (screen* or inventor* or scale* or questio?naire* or assessment* or measurement*).ti,ab,id.
28. questionnaires/ or exp surveys/ or screening/ or exp health screening/ or exp screening tests/
29. 27 or 28
30. 14 and 18 and 25 and 26 and 29
31. limit 30 to (chapter or "comment/reply" or dissertation or editorial or letter or review-book)
32. 30 not 31
33. limit 32 to peer reviewed journal
34. limit 33 to up=20233112-20000101

Pubmed

1. ((("asthma"[Title/Abstract] OR "chronic-illness*"[Title/Abstract] OR "dermatitis"[Title/Abstract] OR "eczema"[Title/Abstract] OR "congenital-heart-disease*"[Title/Abstract] OR "congenital-heart-defect*"[Title/Abstract] OR "diabetes-mellitus-Type-1"[Title/Abstract] OR "diabetes-mellitis-1"[Title/Abstract] OR "inflammatory-bowel-disease*"[Title/Abstract] OR "crohn*"[Title/Abstract] OR "ulcerative-colitis"[Title/Abstract] OR "juvenile-arthritis"[Title/Abstract] OR "cystic-fibrosis"[Title/Abstract] OR "bronchiectasis"[Title/Abstract] OR "haemophilia"[Title/Abstract] OR "hemophilia"[Title/Abstract] OR "bone-disease*"[Title/Abstract] OR "ambulatory care"[Title/Abstract] OR "outpatient clinics"[Title/Abstract] OR "hospital clinic"[Title/Abstract] OR "autoimmune-disease*"[Title/Abstract] OR "auto-immune-disease*"[Title/Abstract] OR "kidney-failure*"[Title/Abstract] OR "immediate-hypersensitivit*"[Title/Abstract] OR "immediate-hyper-sensitivit*"[Title/Abstract]) AND

2. ("parent*"[Title/Abstract] OR "mother*"[Title/Abstract] OR "maternal"[Title/Abstract] OR "father*"[Title/Abstract] OR "paternal"[Title/Abstract] OR "caregiver*"[Title/Abstract] OR "care-giver*"[Title/Abstract] OR "guardian*"[Title/Abstract]) AND

3. ("Anxiety"[Title/Abstract] OR "anxious*"[Title/Abstract] OR "depress*"[Title/Abstract] OR "distress"[Title/Abstract] OR "mental-health"[Title/Abstract] OR "stress"[Title/Abstract] OR "caregiver-burden"[Title/Abstract] OR "mental-disorder*"[Title/Abstract] OR "mental-disease*"[Title/Abstract] OR "burnout"[Title/Abstract] OR "psychological-wellbeing"[Title/Abstract]) AND

4. ("newborn*"[Title/Abstract] OR "new-born*"[Title/Abstract] OR "baby"[Title/Abstract] OR "babies"[Title/Abstract] OR "neonat*"[Title/Abstract] OR "neo-nat*"[Title/Abstract] OR "infan*"[Title/Abstract] OR "toddler*"[Title/Abstract] OR "pre-schooler*"[Title/Abstract] OR "preschooler*"[Title/Abstract] OR "kinder"[Title/Abstract] OR "kinders"[Title/Abstract] OR "kindergarten*"[Title/Abstract] OR "boy"[Title/Abstract] OR "boys"[Title/Abstract] OR "girl"[Title/Abstract] OR "girls"[Title/Abstract] OR "child"[Title/Abstract] OR "children"[Title/Abstract] OR "childhood"[Title/Abstract] OR "pediatric*"[Title/Abstract] OR "paediatric*"[Title/Abstract] OR "school-age*"[Title/Abstract] OR "schoolage*"[Title/Abstract] OR "schoolchild*"[Title/Abstract] OR "schoolgirl*"[Title/Abstract] OR "schoolboy*"[Title/Abstract] OR "adolescen*"[Title/Abstract] OR "youth"[Title/Abstract] OR "youths"[Title/Abstract] OR "teen"[Title/Abstract] OR "teens"[Title/Abstract] OR "teenage*"[Title/Abstract]) AND

5. ("screen*"[Title/Abstract] OR "inventor*"[Title/Abstract] OR "scale*"[Title/Abstract] OR "questionaire*"[Title/Abstract] OR "questionnaire*"[Title/Abstract] OR "assessment*"[Title/Abstract] OR "measurement*"[Title/Abstract] OR "survey*"[Title/Abstract]) AND

6. (NOTNLM OR publisher[sb] OR inprocess[sb] OR pubmednotmedline[sb] OR indatareview[sb] OR pubstatusaheadofprint)) AND

7. (2000:2023[pdat])) NOT

8. ((("asthma"[Title/Abstract] OR "chronic-illness*"[Title/Abstract] OR "dermatitis"[Title/Abstract] OR "eczema"[Title/Abstract] OR "congenital-heart-disease*"[Title/Abstract] OR "congenital-heart-defect*"[Title/Abstract] OR "diabetes-mellitus-Type-1"[Title/Abstract] OR "diabetes-mellitis-1"[Title/Abstract] OR "inflammatory-bowel-disease*"[Title/Abstract] OR "crohn*"[Title/Abstract] OR "ulcerative-colitis"[Title/Abstract] OR "juvenile-arthritis"[Title/Abstract] OR "cystic-fibrosis"[Title/Abstract] OR "bronchiectasis"[Title/Abstract] OR "haemophilia"[Title/Abstract] OR "hemophilia"[Title/Abstract] OR "bone-disease*"[Title/Abstract] OR "ambulatory care"[Title/Abstract]

OR "outpatient clinics"[Title/Abstract] OR "hospital clinic"[Title/Abstract] OR "autoimmune-disease*"[Title/Abstract] OR "auto-immune-disease*"[Title/Abstract] OR "kidney-failure*"[Title/Abstract] OR "immediate-hypersensitivit*"[Title/Abstract] OR "immediate-hyper-sensitivit*"[Title/Abstract]) AND

9. ("parent*"[Title/Abstract] OR "mother*"[Title/Abstract] OR "maternal"[Title/Abstract] OR "father*"[Title/Abstract] OR "paternal"[Title/Abstract] OR "caregiver*"[Title/Abstract] OR "care-giver*"[Title/Abstract] OR "guardian*"[Title/Abstract]) AND ("Anxiety"[Title/Abstract] OR "anxious*"[Title/Abstract] OR "depress*"[Title/Abstract] OR "distress"[Title/Abstract] OR "mental-health"[Title/Abstract] OR "stress"[Title/Abstract] OR "caregiver-burden"[Title/Abstract] OR "mental-disorder*"[Title/Abstract] OR "mental-disease*"[Title/Abstract] OR "burnout"[Title/Abstract] OR "psychological-wellbeing"[Title/Abstract]) AND

10. ("newborn*"[Title/Abstract] OR "new-born*"[Title/Abstract] OR "baby"[Title/Abstract] OR "babies"[Title/Abstract] OR "neonat*"[Title/Abstract] OR "neo-nat*"[Title/Abstract] OR "infan*"[Title/Abstract] OR "toddler*"[Title/Abstract] OR "pre-schooler*"[Title/Abstract] OR "preschooler*"[Title/Abstract] OR "kinder"[Title/Abstract] OR "kinders"[Title/Abstract] OR "kindergarten*"[Title/Abstract] OR "boy"[Title/Abstract] OR "boys"[Title/Abstract] OR "girl"[Title/Abstract] OR "girls"[Title/Abstract] OR "child"[Title/Abstract] OR "children"[Title/Abstract] OR "childhood"[Title/Abstract] OR "pediatric*"[Title/Abstract] OR "paediatric*"[Title/Abstract] OR "school-age*"[Title/Abstract] OR "schoolage*"[Title/Abstract] OR "schoolchild*"[Title/Abstract] OR "schoolgirl*"[Title/Abstract] OR "schoolboy*"[Title/Abstract] OR "adolescen*"[Title/Abstract] OR "youth"[Title/Abstract] OR "youths"[Title/Abstract] OR "teen"[Title/Abstract] OR "teens"[Title/Abstract] OR "teenage*"[Title/Abstract]) AND

11. ("screen*"[Title/Abstract] OR "inventor*"[Title/Abstract] OR "scale*"[Title/Abstract] OR "questionaire*"[Title/Abstract] OR "questionnaire*"[Title/Abstract] OR "assessment*"[Title/Abstract] OR "measurement*"[Title/Abstract] OR "survey*"[Title/Abstract]) AND

12. (NOTNLM OR publisher[sb] OR inprocess[sb] OR pubmednotmedline[sb] OR indatareview[sb] OR pubstatusaheadofprint)) AND ((booksdocs[Filter] OR casereports[Filter] OR comment[Filter] OR editorial[Filter] OR guideline[Filter] OR letter[Filter] OR practiceguideline[Filter]) AND

CINAHL

S33 s31 not s32 AND EM 20233112-20000101
S32 S14 AND S19 AND S26 AND S27 AND S30
S31 S14 AND S19 AND S26 AND S27 AND S30
S30 S28 OR S29
S29 (MH "Questionnaires+") or (MH "Health Screening")
S28 TI (screen* or inventor* or scale* or questio#naire* or assessment* or measurement*) OR AB (screen* or inventor* or scale* or questio#naire* or assessment* or measurement*)
S27 TX newborn* or new-born* or baby or babies or neonat* or neo-nat* or infan* or toddler* or pre-schooler* or preschooler* or kinder or kinders or kindergarten* or kinder-aged or boy or boys or girl or girls or child or children or childhood or pediatric* or paediatric* or school-age* or schoolage* or schoolchild* or schoolgirl* or schoolboy* or adolescen* or youth or youths or teen or teens or teenage*
S26 S20 OR S21 OR S22 OR S23 OR S24 OR S25
S25 TI (Anxiety or anxious* or depress* or distress or mental-health or stress or caregiver-burden or mental-disorder* or mental-disease*) or AB (Anxiety or anxious* or depress* or distress or mental-health or stress or caregiver-burden or mental-disorder* or mental-disease*)
S24 (MH "Stress, Psychological")
S23 (MH "Caregiver Burden")
S22 (MH "Depression+")
S21 (MH "Anxiety") OR (MH "Anxiety Disorders+")
S20 (MH "Mental Health")
S19 S15 OR S16 OR S17 OR S18
S18 TI (parent* or mother* or maternal or father* or paternal or caregiver* or care-giver* or guardian*) or AB (parent* or mother* or maternal or father* or paternal or caregiver* or care-giver* or guardian*)
S17 (MH "Guardianship, Legal")
S16 (MH "Caregivers")
S15 (MH "Parents+")
S14 S1 OR S2 OR S3 OR S4 OR S5 OR S6 OR S7 OR S8 OR S9 OR S10 OR S11 OR S12 OR S13
S13 TI (asthma or chronic-illness* or dermatitis or eczema or congenital-heart-disease* or congenital-heart-defect* or diabetes-mellitus-Type-1 or diabetes-mellitis-1 or inflammatory-bowel-disease* or crohn* or ulcerative-colitis or juvenile-arthritis or cystic-fibrosis or bronchiectasis or haemophilia or hemophilia or kidney-failure* or immediate-hypersensitivit* or immediate-hyper-sensitivit*) OR AB (asthma or chronic-illness* or dermatitis or eczema or congenital-heart-disease* or congenital-heart-defect* or diabetes-mellitus-Type-1 or diabetes-mellitis-1 or inflammatory-bowel-disease* or crohn* or ulcerative-colitis or juvenile-arthritis or cystic-fibrosis or bronchiectasis or haemophilia or hemophilia or kidney-failure* or immediate-hypersensitivit* or immediate-hyper-sensitivit*)
S12 (MH "Kidney Failure, Chronic") or (MH "Renal Tubular Transport, Inborn Errors") or (MH "Hypersensitivity, Immediate") Search modes- Boolean/Phrase Interface- EBSCOhost Research
S11 (MH "Bronchiectasis") or (MH "Cystic Fibrosis")
S10 (MH "Ambulatory Care") OR (MH "Ambulatory Care Facilities") or (MH "Outpatient Service")
S9 (MH "Bone Diseases, Endocrine+")
S8 (MH "Arthritis, Juvenile Rheumatoid")
S7 (MH "Hemophilia B") OR (MH "Hemophilia")
S6 (MH "Inflammatory Bowel Diseases+")
S5 (MH "Diabetes Mellitus, Type 1")
S4 (MH "Heart Defects, Congenital")
S3 (MH "Dermatitis, Atopic") or (MH "Eczema")

S2 (MH "Asthma") OR (MH "Asthma, Exercise-Induced")
S1 (MH "Chronic Disease+")
